# Gender differences in empathy, compassion, and prosocial donations, but not theory of mind in a naturalistic social task

**DOI:** 10.1038/s41598-023-47747-9

**Published:** 2023-11-25

**Authors:** Brennan McDonald, Philipp Kanske

**Affiliations:** Clinical Psychology and Behavioral Neuroscience, Faculty of Psychology, Technische Universität Dresden, Chemnitzer Straße 46, 01187 Dresden, Germany

**Keywords:** Psychology, Human behaviour

## Abstract

Despite broad interest, experimental evidence for gender differences in social abilities remains inconclusive. Two important factors may have limited previous results: (i) a lack of clear distinctions between empathy (sharing another's feelings), compassion (a feeling of concern toward others), and Theory of Mind (ToM; inferring others’ mental states), and (ii) the absence of robust, naturalistic social tasks. Overcoming these limitations, in Study 1 (N = 295) we integrate three independent, previously published datasets, each using a dynamic and situated, video-based paradigm which disentangles ToM, empathy, and compassion, to examine gender differences in social abilities. We observed greater empathy and compassion in women compared to men, but found no evidence that either gender performed better in ToM. In Study 2 (n = 226) we extend this paradigm to allow participants to engage in prosocial donations. Along with replicating the findings of Study 1, we also observed greater prosocial donations in women compared to men. Additionally, we discuss an exploratory, novel finding, namely that ToM performance is positively associated with prosocial donations in women, but not men. Overall, these results emphasize the importance of establishing experimental designs that incorporate dynamic, complex stimuli to better capture the social realities that men and women experience in their daily lives.

## Introduction

Taking others’ perspective and sharing their emotions are crucial abilities underlying successful social interactions. Equally important are compassionate feelings for others, linked with the motivation to help^1^. Neuroimaging and behavioral data have begun to show that the social affective states of *compassion* (caring feeling for another^1^) and *empathy* (sharing emotions with another^2^), while conceptually connected, are discrete processes at the neural level^1,3^. Further separated from social affect is the social cognitive process of mentalizing or *Theory of Mind* (ToM), involving the abstract, propositional representation of others’ mental states^4,5^.

A growing body of evidence shows that significant inter-individual differences exist between these distinct social abilities^6^. However, the evidence for inter-individual gender differences in these social capacities is inconclusive. Numerous studies have reported an advantage for women in emotional face recognition^7^, self-reports of empathy/compassion^8–10^, and affective understanding of others^11–13^. Conversely, other investigations yield ambiguous findings^14–16^, observe extremely small effect sizes in large samples^8^, or find no gender differences in social abilities^17–19^. Regarding prosocial behaviour, results again appear dependent on the sample, situation, or definitions of prosocial behaviour^20^. Evidence does suggest women are often more prosocial compared to men^21,22^. However, several studies also show men are more altruistic than women in situations involving strangers, requiring the helper’s initiative rather than responding to a request for help^23,24^.

A possible explanation for these inconsistent findings may be a narrow focus on isolated components of social processing (e.g. focusing on self-reports of empathy) or using unrelated measurement tools (e.g. comparing empathy and ToM measured by two unrelated tasks) leading many studies to inadequately distinguish between social affect and social cognition^9^. However, as these social processes are claimed to be separable^25,26^, it is important that measurement is performed within the same sample and paradigm to allow for gender-specific differences to be distinguished. Additionally, the experimental designs used to evoke social responses play a crucial role in accurately capturing real-world social functioning and can strongly influence observed results. To date, the majority of research examining gender differences in social abilities rely on static measures, often requiring responses to isolated stimuli (e.g., videos/images of postures, eyes, faces, or individual body-parts), self-report questionnaires, or tasks via a computer with no obvious social partner. By using more ecologically valid social stimuli that are dynamic, multimodal and situated – and thus better reflecting the complexity of real-world social behaviour – inter-individual differences in social affect and social cognition may be more accurately captured, including potential differences between men and women. For the current studies, we asked if gender differences in empathy, compassion, ToM and prosocial donations are observable during naturalistic, dynamic, video-based social situations.

For Study 1, we combined the results of three independent, previously published experimental datasets each using the EmpaToM task (N = 295), a validated assessment of social affect and social cognition^3^. The task involves 48 videos displaying either neutral or emotionally negative narrations. Following each video, participants’ empathic responding, compassion, and ToM performance were measured. The EmpaToM task thus provides the unique advantage of allowing social affect and social cognition to be assessed near simultaneously with respect to the same stimuli. Additionally, because the EmpaToM task uses video narratives, participants are exposed to situated, dynamic and multimodal social stimuli, including the narrator’s autobiographical story, body language, facial expressions and vocal inflections, increasing the ecological validity of the social measures.

For Study 2, we utilized an additional independent dataset (N = 226), which included an extended version of the EmpaToM task with a novel measure of prosociality/charitable donations. Along with allowing for a replication of Study 1, this additional measure allowed for empathy, compassion and ToM to be directly correlated with each participant’s willingness to engage in prosocial behavior.

Based on previous evidence suggesting a social affect advantage for women, we hypothesized the following: women will show greater affect sharing (i.e., empathy) and report greater compassion during the emotionally negative narrations compared to men. Regarding ToM, the evidence is far less clear whether gender differences exist. Thus, we aimed to test whether a gender advantage exists in ToM performance. With respect to the prosocial donation measure in Study 2, we hypothesized based on previous evidence that women would show greater willingness to engage in prosocial donations compared to men. Finally, we examined if social affect and social cognition measures correlated with prosocial donations, both across the sample and for each gender. We then explored subsequent findings of interest by testing if significant measures were associated with participant’s prosocial donations and if gender differences existed in this regard.

## Results: study 1

### Gender differences in empathy and compassion

While controlling for differences between the experimental locations (MRI scanner or laboratory) and participant’s age, we analysed the effects of gender differences on empathy and compassion using a mixed design, repeated measures ANCOVA with gender (men vs. women) as the between-subject factor and video emotionality (emotionally negative vs. neutral videos) as the within-subject factor. Differences regarding empathy and compassion would emerge as an interaction effect between participants’ gender and the emotionality of the video narrations. Effect size is reflected by the partial eta squared (η^2^p).

**Figure 1:**
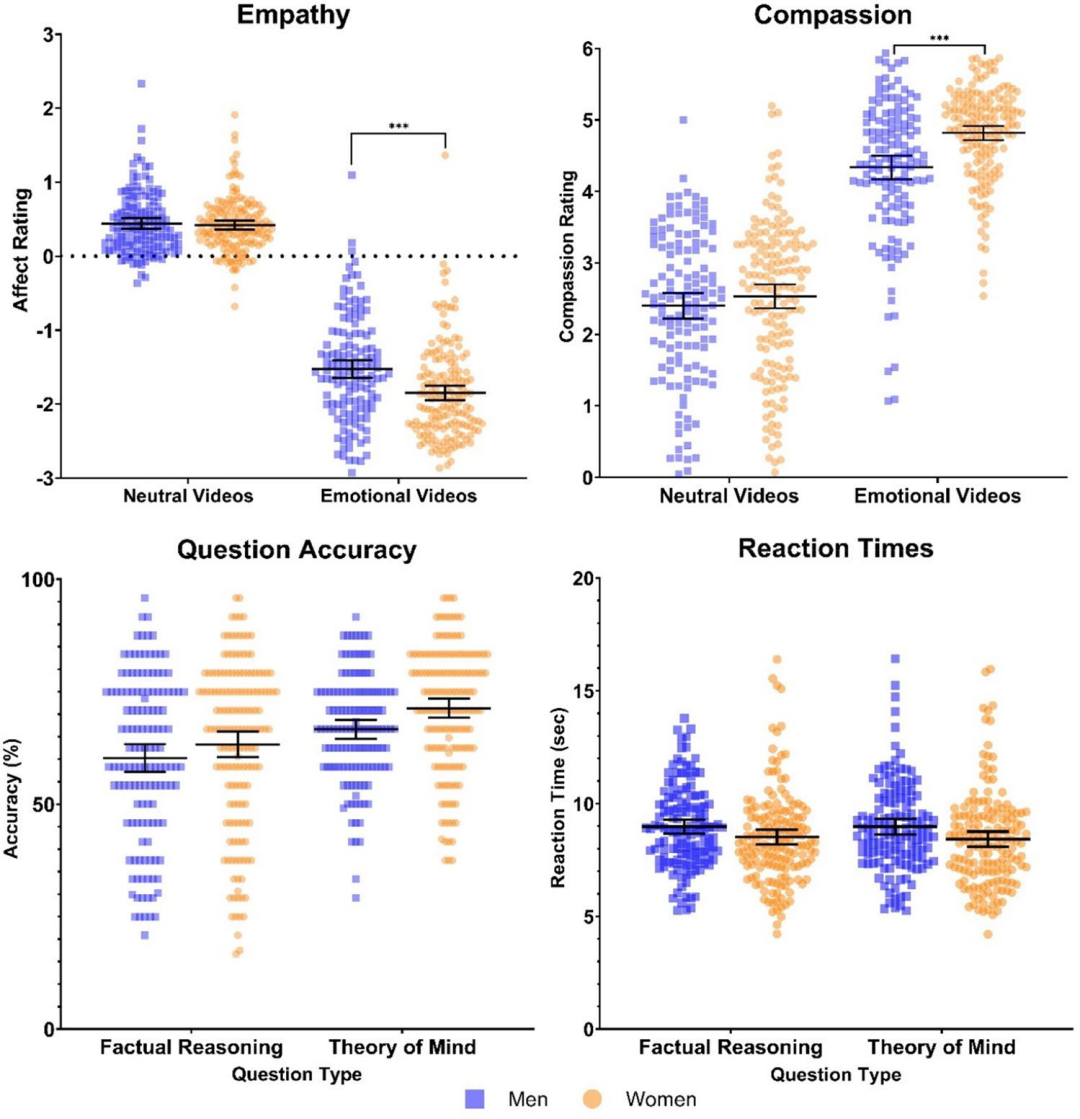
***p < 0.001. Study 1: Empathy ratings showed that emotionally negative videos elicited stronger negative affect compared to affect ratings after neutral videos. Significant interaction effects were observed between emotional video narrations and participant’s gender for empathy and compassion measures. Analysis of question accuracy and reaction times (on correctly answered trials) revealed no significant interaction effects among theory of mind questions and participants’ gender. Bars indicate mean ± 95% CI.

The analyses showed a significant main effect of video emotionality for participants’ ratings of the valence of their own affect (F(1, 291) = 358.874, p < 0.001, η^2^p = 0.55) and for participant’s compassion ratings (F(1, 291) = 328.242, p < 0.001, η^2^p = 0.53), indicating that emotionally negative videos elicited stronger negative affect (i.e., empathic emotion sharing) and stronger compassion than neutral videos. Additionally, a main effect of participants’ gender on affective valence (F(1, 291) = 11.845, p < 0.001, η^2^p = 0.039) and compassion (F(1, 292) = 10.382, p = 0.001, η^2^p = 0.034) was observed. Specifically, women reported stronger overall negative affect and compassion than men. However, we also observed significant interaction effects between participants’ gender and video emotionality for both empathy (F(1, 291) = 9.364, p = 0.002, η^2^p = 0.031) and compassion (F(1, 291) = 10.382, p = 0.001, η^2^p = 0.034). Post-hoc, paired t-tests confirmed the hypotheses: Following emotionally negative narrations, empathy (t(293) = 4.104, p < 0.001, d = 0.67) and compassion (t(293) = 5.126, p < 0.001, d = 0.80) were significantly stronger in women than in men (see Fig. 1).

### Gender differences in theory of mind

To test for gender differences in ToM performance, we analysed accuracy and reaction times in the multiple-choice questions, using a repeated measures ANCOVA with question type (factual-reasoning vs. ToM) as the within-subject factor and participants’ gender (men vs. women) as the between-subject factor, whilst controlling for differences between the experimental locations (MRI scanner or laboratory) and participant’s age. Gender differences in social cognition would emerge as an interaction effect between question type and participants’ gender with increased accuracy and shorter reaction times indicating better ToM performance.

The analyses showed no significant main effect for question type with respect to accuracy (F(1, 291) = 1.636, p = 0.20, η2p = 0.006). However, a significant main effect of question type was observed for reaction times (F(1, 291) = 10.229, p < 0.002, η2p = 0.034) with participants demonstrating shorter reaction times for ToM questions, indicating that ToM questions were slightly easier than the factual reasoning questions, as observed previously for this task^3,27^. No main effect of gender was observed for reaction times (F(1, 291) = 4.801, p = 0.02, η2p = 0.016), however, a main effect of gender was observed for question accuracy (F(1, 291) = 3.036, p = 0.08, η2p = 0.01) with women demonstrating greater overall accuracy for both question types. Finally, the analyses revealed no significant interaction effect between participants’ gender and question type for both accuracy (F(1, 291) = 1.282, p = 0.25, η2 = 0.004) and reaction times (F(1, 291) = 0.565, p = 0.45, η2p = 0.002). Thus, we found no evidence for gender differences in ToM performance (see Fig. 1).

## Results: study 2

### Gender differences in empathy and compassion

**Figure 2:**
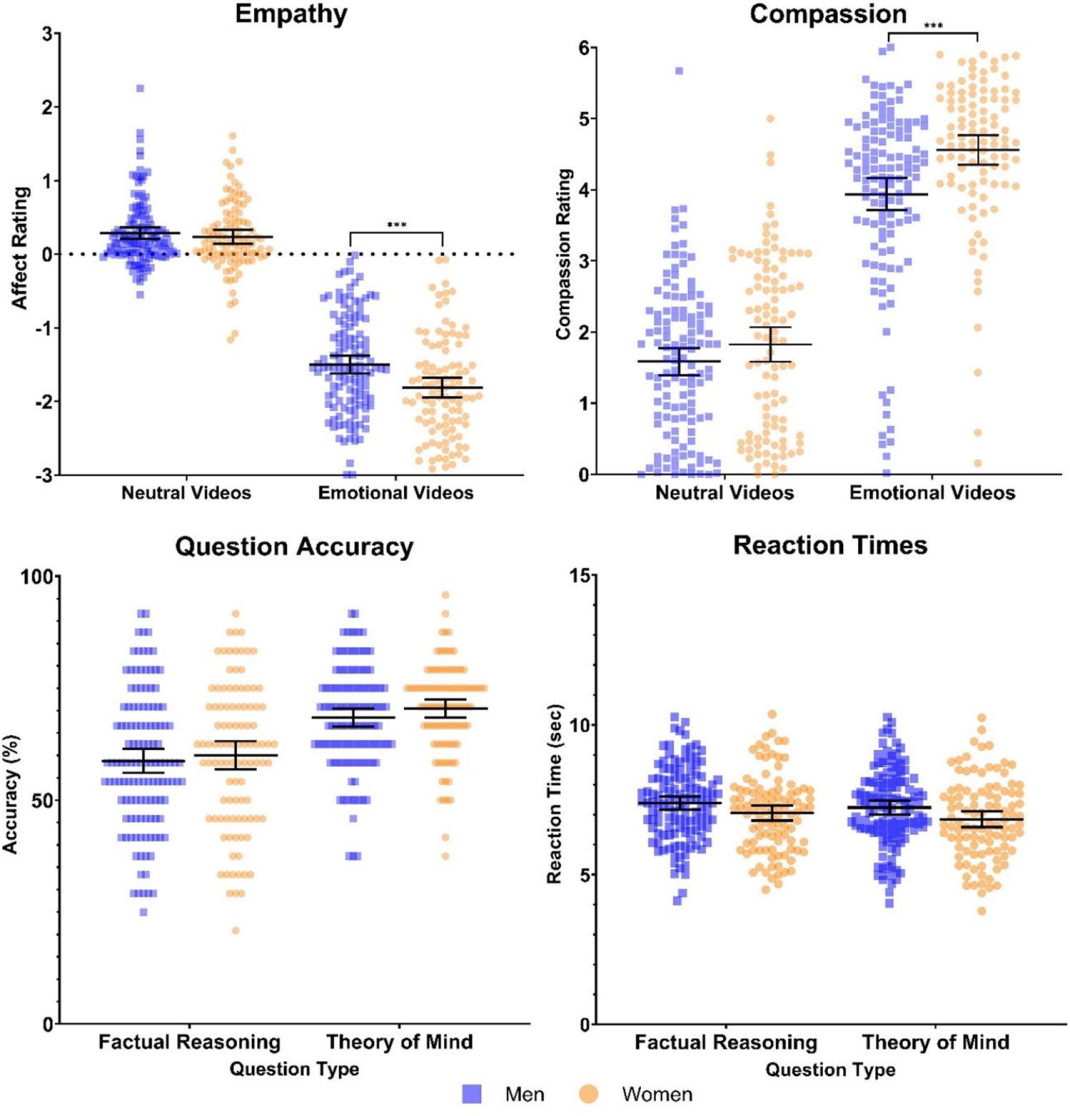
***p < 0.001. Study 2: Replicating the results of Study 1 in an online environment, empathy ratings showed that emotionally negative videos elicited stronger negative affect compared to affect ratings after neutral videos. Significant interaction effects were observed between emotional video narrations and participant’s gender for empathy and compassion measures. Analysis of question accuracy and reaction times (on correctly answered trials) revealed no significant interaction effects among theory of mind questions and participants’ gender. Bars indicate mean ± 95% CI.

We analysed the effects of gender differences on empathy and compassion using a repeated measures ANCOVA with gender (men vs. women) as the between-subject factor and video emotionality (emotionally negative vs. neutral videos) as the within-subject factor, controlling for participant’s age. Differences regarding empathy and compassion would emerge as an interaction effect between participants’ gender and the emotionality of the video narrations. The analyses for Study 2 showed a significant main effect of video emotionality for participants’ ratings of the valence of their own affect (F(1, 223) = 87.505, p < 0.001, η2p = 0.28) and for participant’s compassion ratings (F(1, 223) = 86.265, p < 0.001, η2p = 0.27), indicating that emotionally negative videos elicited stronger negative affect (i.e., empathic emotion sharing) and stronger compassion than neutral videos. Additionally, a main effect of participants’ gender on affective valence (F(1, 223) = 17.013, p < 0.001, η2p = 0.07) and compassion (F(1, 223) = 9.579, p = 0.002, η2p = 0.041) was observed. Specifically, women reported stronger overall negative affect and compassion than men. However, we also observed significant interaction effects between participants’ gender and video emotionality for both empathy (F(1, 223) = 4.298, p < 0.03, η2p = 0.019) and compassion (F(1, 223) = 5.235, p = 0.02, η2p = 0.023). Post-hoc, paired t-tests confirmed the hypotheses: Following emotionally negative narrations, empathy (t(225) = 3.493, p < 0.001, d = 0.46) and compassion (t(225) = 3.948, p < 0.001, d = 0.52) were significantly stronger in women than in men (see Fig. 2).

### Gender differences in theory of mind

To test for gender differences in ToM performance, we analysed accuracy and reaction times in the multiple-choice questions, using a repeated measures ANCOVA with question type (factual-reasoning vs. ToM) as the within-subject factor and participants’ gender (men vs. women) as the between-subject, controlling for participant’s age. Gender differences in social cognition would emerge as an interaction effect between question type and participants’ gender with increased accuracy and shorter reaction times indicating better ToM performance. The analyses showed a significant main effect for question type with participants demonstrating higher accuracy (F(1, 223) = 12.065, p < 0.001, η2p = 0.051) for ToM questions, indicating that ToM questions were slightly easier than the factual reasoning questions, as observed previously for this task^3,27^. No main effect for question type was observed with respect to reaction times (F(1, 223) = 1.457, p = 0.22, η2p = 0.05). No main effect of gender was observed for question accuracy (F(1, 223) = 1.364, p = 0.24, η2p = 0.006), while a main effect of gender was observed for question reaction times (F(1, 223) = 5.504, p = 0.02, η2p = 0.024) with women demonstrating overall shorter reaction times for both question types. Finally, the analyses revealed no significant interaction effect between participants’ gender and question type for both accuracy (F(1, 223) = 0.185, p = 0.66, η2p = 0.001) and reaction times (F(1, 223) = 1.457, p = 0.50, η2 = 0.002). Thus, we found no evidence for gender differences in ToM performance (see Fig. 2).

### Gender differences in prosocial donations

**Figure 3:**
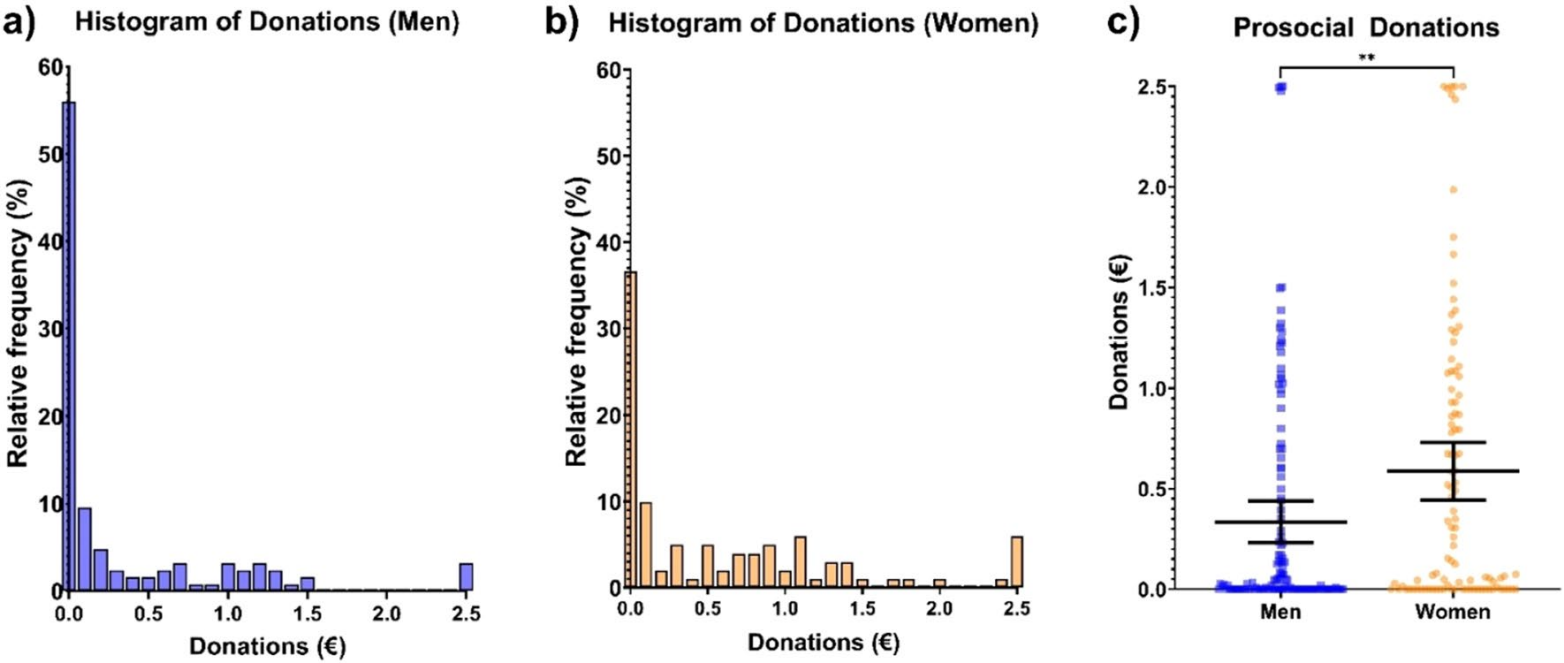
**p < 0.01. (**a**) and (**b**) Histograms showing the relative frequency of the average donations for men (n = 125) and women (n = 101) with a centre bin of 0.1. (**c**) Significant difference observed between the average donation amounts in women compared to men. Bars indicate mean ± 95% CI.

Utilizing the prosocial donation data from Study 2 (n = 226, 101 women), we next tested if there were gender differences in prosocial donations. Due to the relatively large number of participants, who did not donate at all (men: n = 54, women: n = 29), the prosocial data was zero inflated with a right tailed distribution (men: skewness = 2.119, women: skewness = 1.304). Thus, the assumptions for an independent t-test analysis were not met. As alternatives that better fit the data distribution we employed a Mann–Whitney U test and a two-sample Kolmogorov–Smirnov test. The two-sample Kolmogorov–Smirnov test indicated that the cumulative distribution for donations by women differs from the cumulative distribution for donations by men (D = 0.225, p = 0.007, see Fig. 3a and b). The Mann–Whitney U test revealed that women (Md = 0.30, n = 101) were willing to donate significantly more money on average to a charitable cause than men (Md = 0.02, n = 125), U = 4859, z = -3.052, p = 0.002, with a small effect size r = 0.20 (see Fig. 3c.).

### Raw correlations between prosocial donations and empathy, compassion and theory of mind

To explore whether a possible relationship exists between social affect/cognition and prosocial donations we calculated correlation coefficients for Study 2, both for the total sample and for each gender (see Table 1). Because the prosocial donation data violated the normality assumption required for the calculation of Pearson’s r, the association between measures was examined using a nonparametric statistic (Spearman’s ρ). To enable the correlation of inter-individual differences, the following individual averages were used. The ability to empathically share others’ emotion is best reflected in participants’ reported affect after the emotionally negative narrations, capturing their affective state after viewing another person in distress. Because compassion and prosocial donations are positive social attributes regardless of video content, these measures reflect participants’ average value across all narrations (as done previously^28^). ToM accuracy and factual-reasoning accuracy are the percentage of questions answered correctly for each question type. Reaction times reflect the time taken to answer either ToM or factual reasoning questions on those trials where the answer given was correct.

**Table 1:** Inter-scale correlations (Spearman’s ρ) for measures of Social Affect, Social Cognition, and Prosocial Donations.

	1	2	3	4	5	6	7
1. Empathy	–	− 0.56* − 0.49* (− 0.60*)	− 0.17* − 0.06 (− 0.22*)	− 0.21* − 0.14 (− 0.20*)	** − 0.00** − 0.02 (− 0.09)	− 0.25* − 0.19 (− 0.28*)	** − 0.05** − 0.07 (− 0.14)
2. Compassion		–	0.33* 0.35* (0.25*)	**0.03** 0.08 (− 0.07)	0.13* 0.21* (0.12)	**0.10** 0.03 (0.15)	0.17* 0.25* (0.17*)
3. Prosocial donation			–	**0.00** 0.23* (− 0.21*)	**0.08** 0.11 (0.09)	**0.06** 0.14 (− 0.03)	**0.11** 0.09 (0.15)
4. ToM accuracy				–	** − 0.11** − 0.19 (-0.02)	0.46* 0.47* (0.43*)	− 0.16* − 0.16 (− 0.15)
5. ToM reaction time					–	** − 0.07** − 0.19* (0.04)	0.83* 0.87* (0.79*)
6. Factual reasoning accuracy						–	** − 0.11** − 0.14 (− 0.07)
7. Factual reasoning reaction time							–

*p < 0.05, Coefficients in bold represent the entire sample. Coefficients not in bold are for women only (n = 101). Coefficients for men reported in parentheses (n = 125).

#### Full sample

 Both empathy and compassion correlated significantly with prosocial donations. Neither ToM accuracy/reaction times nor factual-reasoning accuracy/reaction times showed significant correlations with prosocial donations.

#### Gender differences

**Figure 4:**
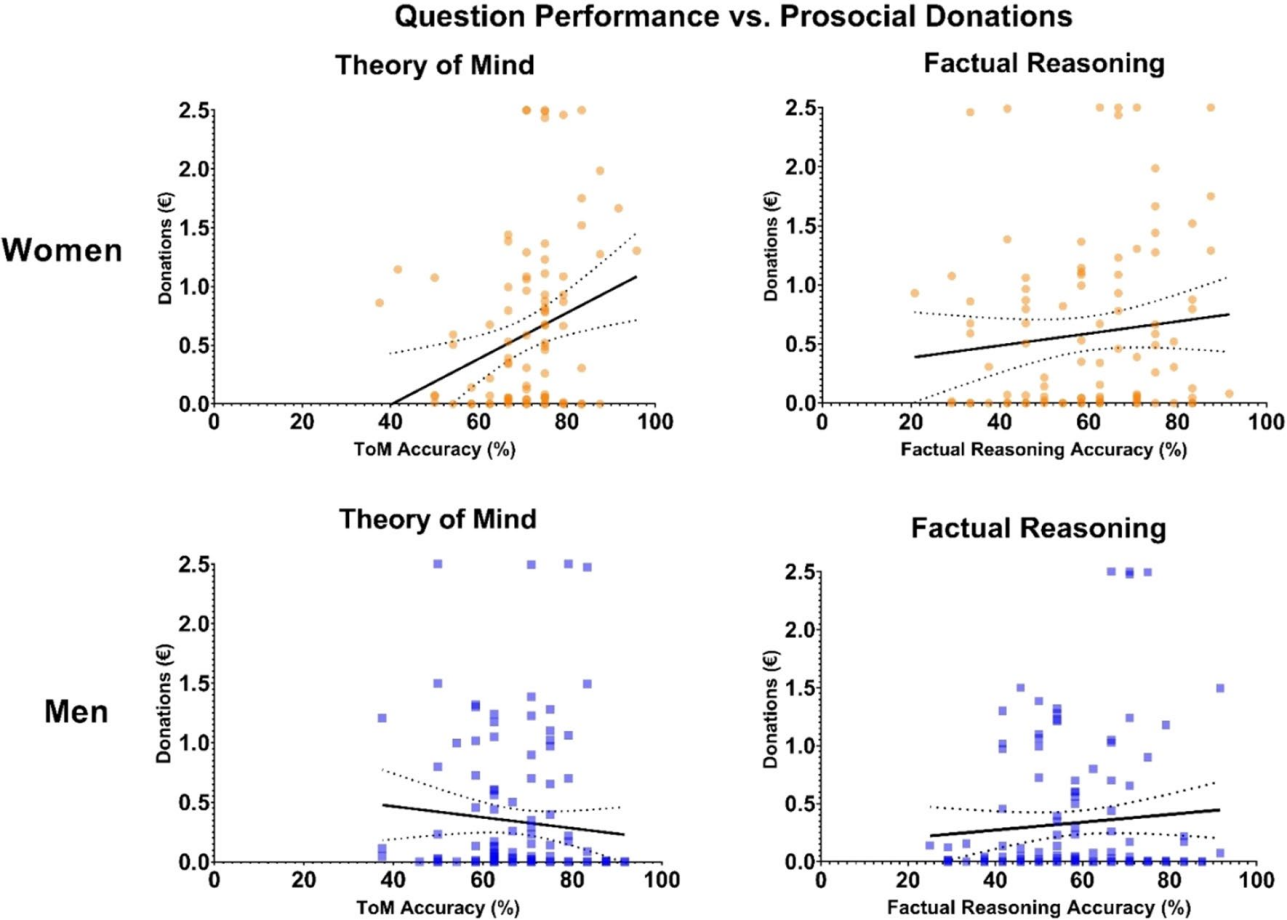
Scatterplots displaying linear correlations between ToM accuracy vs. average donation and factual reasoning accuracy vs. average donation for men (n = 125) and women (n = 101). Dotted curves indicate the 95% confidence bands of the best-fit line. ToM accuracy showed a significant positive correlation with prosocial donations in women (ρ = 0.23) and a significant negative correlation in men (ρ =  − 0.21). Factual accuracy did not correlate significantly.

 On inspection, the only correlation coefficients that clearly differed between men and women were that of ToM accuracy vs. prosocial donations with women showing a significant positive correlation and men showing a significant negative correlation (see Fig. 4.). To test if these correlation coefficients significantly diverged, we performed a Fisher's *z*-transformation. This method is sufficient for testing differences between Spearman’s ρ coefficients^29^. The analysis revealed that for ToM accuracy vs. prosocial donations the correlation coefficient for women differed significantly from that of men (| ρ_diff_ |= 0.44, Z = 3.298, p < 0.001). No other gender difference between correlation coefficients was statistically significant (p > 0.05).

### Prosocial donations and ToM performance

Finally, to explore the possible factors influencing the observed divergent correlations between the average ToM performance and prosocial donations in men and women we used a generalized linear model with a gamma distribution and logarithmic mean function expand for repeated measures (as a robust alternative to a linear regression model with distributional assumptions that better fit the data). We also performed the analysis with the robust sandwich method to estimate the standard errors in these models avoiding the assumption that the model correctly specifies the variances of the residuals.

This data-driven model was used to test if the interaction between ToM accuracy and participants’ gender was significantly associated with prosocial donations, controlling for participants’ factual reasoning accuracy, age and highest level of education. Note: a Chi-square test revealed no gender difference in the level of education (X^2^ [df = 2, N = 226] = 1.264, p = 0.53). Level of education was included as a categorical, nominal data in this model. Level of education was included in the model due to the positive association between educational attainment and earning potential^30^. We also included the interaction term factual-reasoning accuracy x gender to control for the more general influence of question performance and participants’ gender on donations.

**Figure 5:**
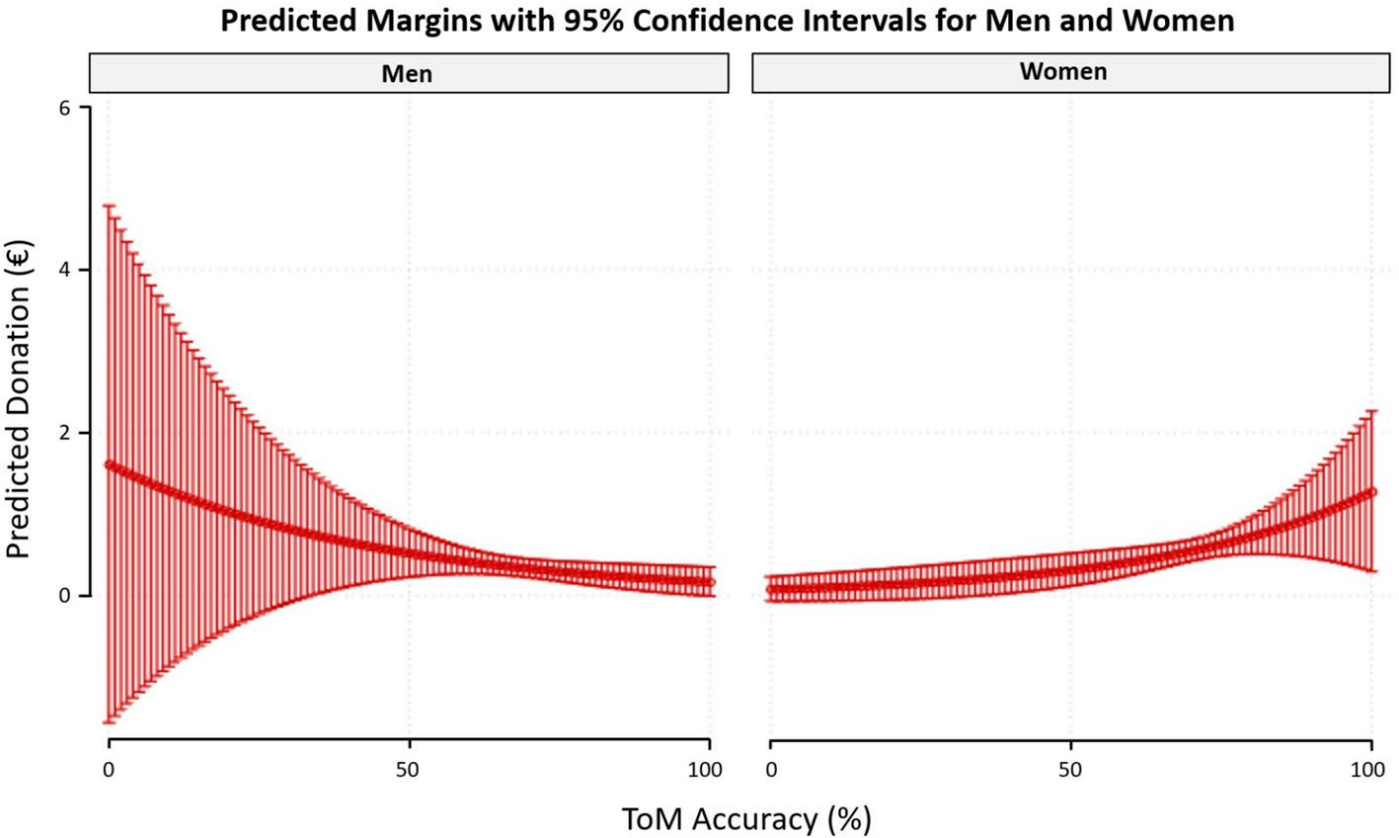
Predicted margins with 95% confidence intervals displaying the regression curves for ToM accuracy vs. average donation for men (n = 125) and women (n = 101), controlling for factual reasoning accuracy, age, and level of education. ToM accuracy significantly predicts donations in women and does not predict donations in men.

The full results of the model are available in the supplementary data. We found a significant interaction effect for ToM accuracy vs. participants’ gender (β = 1.05, p = 0.013) and no significant interaction for factual reasoning accuracy vs. participants’ gender (β = 0.978, p > 0.05). Specifically, ToM accuracy significantly predicted a 2.8% (95% CI = 0.1% to 5.5%) increase in donations per percentage point in women (see Fig. 5.). We found no evidence that ToM performance significantly predicts donations in men (a non-significant 2.2% decrease in donations per ToM percentage point with 95% CI = -5.2% to 0.6%). To assess goodness of fit, Akaike's Information Criterion (AIC) was used to compare the full model and a reduced model (lacking the interaction term of interest: gender x ToM accuracy). Following Arnold^31^, lower AIC coefficients were considered to indicate better model fit. We observed a lower AIC for the full model (AIC = 69.14) compared to the reduced model (AIC = 81.51), indicating that the full model better fit the data when including the interaction term of interest.

## Discussion

The current studies investigated gender differences in social affect, social cognition and prosocial donations during naturalistic social narrations. We observed significantly greater empathy, compassion and prosocial donations in women compared to men. By contrast, we found no evidence that either gender performed better in ToM. However, ToM performance was positively associated with prosocial donations in women, but not men. These data expand on previous findings showing an advantage for women in social affective processes and prosocial behaviour, providing additional evidence for inter-individual variation in these separable social abilities.

Various explanations exist as to why social gender differences may occur. For example, evolutionary models propose that variation in reproductive investment generates sexual-selective pressures, differentially favouring the evolution of sex-specific social behaviours^32^. In contrast, gender socialization models posit that cultural beliefs about gender roles influence gender expectations, orienting women and men towards different social behavioural patterns^33,34^.Importantly, both the existence and impact of such gender differences, whether social or biological, are a point of much heated debate^35–39^. For the current studies, we do not commit to any ultimate cause for observed social gender differences. Rather, we focused on examining the possible presence of such differences by addressing several limitations of previous research. Broadly, our goal was to investigate gender differences in relation to two factors: i) the comprehensive, but distinct investigation of social affective and social cognitive processes and ii) the examination of these processes in a robust, naturalistic setting.

Research is beginning to converge on the finding that empathy, compassion and ToM are dissociable processes^25,26^. Additionally, significant intra- and inter-individual differences in these social abilities exist^6^. Despite this, evidence for social gender differences remains unclear. Importantly, as these are claimed to be separable social processes, measurement within the same sample and paradigm is necessary to allow gender differences to be accurately distinguished. Thus, our first goal was to make clear distinctions between empathy, compassion, and ToM, utilizing high-level control conditions (i.e. neutral vs emotional videos and factual-reasoning vs ToM questions). Our second goal was to examine gender differences using stimuli combining numerous aspects of real-life social interactions, thereby overcome some of the limitations of previous research. Specifically, previous investigations examining social gender differences have produced conflicting results regarding whether such differences exist. A reason for these inconsistent findings may be that by over-isolating specific social abilities (e.g., recognizing emotions based on still images of eyes), or having no clear social partner (e.g., indirect behavior on a computer screen) researchers may limit themselves to investigating tiny effect sizes. Indeed, Baez and colleagues^8^ tested gender differences in empathy (i.e. harm of others) using short, animated scenarios of isolated body-parts in a very large sample (n > 10,000) and only found a miniscule effect. However, when taken together, previous findings appear to suggest that women have a slight advantage in detecting affective facial expressions^7^, recognising vocal emotions^40^, and understanding body language/emotional posture^41^. The present studies indicate that by naturalistically combining these discrete social facets in complex, multimodal stimuli of realistic people, robust differences in social affect between the genders can be detected. Specifically, our results exhibit that women have an advantage in empathy and compassion over men.

Regarding our social cognition findings, we did not observe any gender advantage in ToM ability over and above factual-reasoning ability. While evidence suggests that a female advantage in ToM may be present in childhood/adolescence^42–44^, findings with adults are far more mixed, with some results indicating that women may show an advantage on ToM tasks involving an affective component compared to men^11,13,45,46^, while other finding suggest no clear gender differences^9,47–49^. Importantly, as with measures of social affect, these inconsistent results may result from a lack of clear distinctions between the separable processes of social affect and social cognition, thus creating a pronounced degree of overlap between measures. Moreover, this is made additionally confusing by the use of multiple different terms/synonyms in the literature referring to these processes, with social cognition also at times being referred to as cognitive ToM, mentalizing, or cognitive empathy, while empathy has been termed affective ToM or emotional empathy.

Here, we define ToM as the ability to understand and use abstract propositional knowledge about another’s metal states. Although we observed a main effect of gender such that women were more accurate (Study 1) or had shorter reaction times (Study 2) for both question types, we did not observe an interaction effect between question type and gender. This indicates that while women may be better in general at answering the questions during the EmpaToM task, neither gender demonstrate a specific advantage for ToM questions. We interpret this lack of a gender difference as reflecting the complex, cognitive nature of ToM processing. Based on our definition, ToM performance during the EmpaToM task relies on a range of general cognitive abilities including attention, reasoning, memory, and language comprehension, with ToM questions differing from factual-reasoning control questions only in social content. As such, this multifaceted construct relies on the simultaneous application of diverse abilities that fall under the rubric of intelligence. While there exist well-established cognitive gender differences in certain specific tasks^50^, there is no difference in average general intelligence between men and women^51,52^. Our findings expand on this, demonstrating that when using factual-reasoning ability as a high-level control condition, men and women do not differ in their ToM ability. Put another way, the results of the present study provide evidence that neither men nor women have a cognitive advantage in abstract reasoning about another’s mental states during naturalistic situations.

While we found no overall gender difference in social cognition, we did however observe differences in prosocial donations between men and women. Specifically, we found that women, on average, donated more money than men. This is consistent with a large body of work indicating that women are often more prosocial compared to men^21,22^.

Subsequent to this finding, we then explored the linear correlations between prosocial donations and empathy, compassion, ToM and factual reasoning, both at the level of the full sample and between the genders. At the level of the full sample, we observed significant correlations for empathy with prosocial donations, while finding no significant correlations for ToM and factual reasoning. These findings provide support for the empathy-altruism hypothesis^53,54^ and further underline the separable nature of social affective and social cognitive processes^5^. The empathy-altruism hypothesis highlights the strong association between empathy and the motivation to increase the wellbeing of another person^54,55^, with numerous studies supporting the connection between empathy and altruistic behaviour^56–58^. Our results extend these findings by demonstrating that empathy correlates significantly with prosocial donations within the same naturalistic video-based task. Additionally, we also observe a significant association between compassion and prosocial donations, which is also to be expected. Compassion is defined as a feeling of warmth or loving-kindness towards others and is thus understood as an other-directed aspect of social affect, while empathy relates to one’s own (negative) affect in response to the distress of another. In this regard, our data exhibit a stronger correlation for compassion with prosocial decision compared to empathy, consistent with previous data^58^ and the strong motivational component towards others accredited to compassion^59^.

Finally, at the level of the full sample, we observed no significant correlation with ToM and factual reasoning accuracy or RTs. This finding is consistent with the previous observations that affective states represent a more immediate activator of behavioural response than cognitive processes^60^. However, in addition to these findings at the level of the full sample, we also observed gender differences in the linear correlations between prosocial donations and ToM accuracy, with women showing a significant positive correlation and men a significant negative correlation (see Table 1). To examine this result further, we performed an exploratory generalized linear model that better fit the data. This analysis revealed that ToM performance in women, but not men, was positively associated with prosocial donations. Put another way, although we found no evidence for a ToM advantage between men and women, this model suggests that women’s average ToM accuracy is positively associated prosocial donations, controlling for age, level of education, and factual-reasoning performance, with no such associated pattern observed for men.

We speculate that this exploratory, data-driven finding may relate to differences in men and women’s interest in social stimuli. Previous evidence suggests that women tend to show a greater interest/responsiveness to social stimuli when compared to men^7,40^. Additionally, research indicates that both social affective and social cognitive processes are involved in prosocial decision-making^27^. We suggest that an interest in social stimuli may possibly favour the recruitment of social cognition during prosocial decision-making. Put another way, women’s average greater interest in social stimuli compared to men may favour the observed relationship between the ability to take another’s perspective (ToM performance) and respond to another’s needs (prosocial donations). This social interest may also foster women’s willingness to donate more when compared to men, as observed in the current studies. It is important, however, to highlight that this was a data-driven exploratory model based on a single sample and as such requires further replication and conformation before any strong claims can be supported^61^. Future research may thus expand on this exploratory result by replicating this pattern between the genders and probing whether individual differences in social interest mediate the relationship between gender, ToM performance, and prosocial donations.

The current studies also have limitations that warrant mention. First, Study 1 utilized multiple previously published summary datasets, while Study 2 derived from a separate pre-registered investigation. Because the available datasets differed in how demographic data was collected and already had exclusion criteria applied, we chose not to apply our own additional exclusion of participants, beyond those of the original publications. This approach however partially limits the comparability of Study 1 and Study 2 as different approaches are used to define the samples. Second, Study 2 was conducted in an online environment and although participants were requested to undertake the experiment in a quite environment alone, we cannot ensure this was the case. Third, an additional limitation of the current studies is the lack of videos with positive valence. Because the EmpaToM paradigm, to date, does not have validated videos sets with positive valence, we were unable to test if gender differences in social affect and/or ToM are influenced by mirroring, sharing, or understanding positive emotions. However, this is an important facet for understanding social emotions, as previous data suggest that women self-report greater overall negative emotions than men, but show no difference in overall positive emotions^62^. Thus, a fruitful avenue of future investigation will be to extend our findings by including positive valence in social scenarios examining gender differences in empathy, compassion, prosocial donations, and ToM.

In conclusion, our studies examined gender differences using a naturalistic task that allows for clear distinctions between social affect and social cognition. We observed greater empathy, compassion and prosocial donations in women compared to men, but found no evidence that either gender performed better in ToM. However, ToM performance was positively associated with donations in women, but not men in our exploratory, data-driven model. Ultimately, our findings call for both the clear definition of social abilities as well as the use of naturalistic social tasks that incorporate multimodal, dynamic and situated stimuli to better capture the social realities that men and women experience in their daily lives.

## Methods: study 1

Study 1 integrates three previously published, experimental datasets to examine gender differences in social abilities. For each experiment, a version of the EmpaToM task was performed. The datasets were selected based on their availability online with full summary EmpaToM and gender data. Differences and similarities between the datasets are summarized in Table. 1. Dataset 1 (D1) is from a published functional imaging study examining differences in social abilities between young and elderly adults^63^. The data for D1 are available at https://doi.org/10.17605/OSF.IO/7EDBN. Dataset 2 (D2) is from a published functional imaging study examining social abilities and personality^64^. The data for D2 are available at https://osf.io/f7zcr/. Dataset 3 (D3) is from a published study in which the EmpaToM was a baseline measure to investigate meditation practice^58^. The data for D3 is available at https://osf.io/tu2gj/?view_only=f285308c035d451ca2fce5f3788f97e3. The complete dataset and analysis scripts for Study 1 are available at https://osf.io/xgma6/?view_only=d0b7d3e1f10a415fa6c033678de5c9ff. The study protocol for each of the three studies was approved by the Ethics Committee of Technische Universität Dresden (D1: EK-486112015, D2: EK-133042018, D3: EK-180052018). All methods were performed in accordance with the relevant guidelines and regulations. Using G*Power 3^65^, an a priori power analysis estimated a total sample of 200 participants (α = 0.05, 1 – β = 0.8) based on a small effect size (f = 0.1) would be sufficient to detect gender differences. Mann–Whitney U test and ANCOVA analyses were conducted using SPSS (IBM Corp., 2021, Version 28.0.1.0). Graphics were produced using Graphpad Prism (https://www.graphpad.com, Version 9.3.1). This study was not preregistered.

### Participants

In total, 295 participants, consisting of 160 women (age mean = 31.21, SD = 16.84) and 135 men (age mean = 31.96, SD = 17.00), were included in the current analyses. A Mann–Whitney U test indicated that men and women did not differ significantly in age (U = 11,772, p = 0.18). As these previously published datasets were only available as summary data online, with differing demographic measures and already-applied exclusion criteria, no additional layers of exclusions were performed beyond those originally reported in the original publications.

For D1, reported exclusion criteria included excessive or illicit substance use, neurological or cognitive impairment and MRI related reasons. One hundred and one individuals were initially recruited and sixteen individuals were excluded from the final dataset due to MRI related issues (15) or EmpaToM values exceeding three standard deviations of the mean (1), leaving a final data set of 85 participants (43 women). For full details on participants see Stietz and colleagues^63^.

For D2, 168 participants (age: < 18 or > 60 years) were originally invited. Exclusion criteria were MRI related reasons (27), severe mental disorder (3), and not providing sufficient Ecological Momentary Assessment data (16). Thus, 46 participants were excluded yielding a final sample of 122 participants. One participant was additionally excluded from the dataset due to incomplete EmpaToM data, leaving 121 participants (58 women) for the current analysis. For full details see Hildebrandt and colleagues^64^.

For D3, participants were recruited through local advertisements via flyer or e-mail for a study examining meditation practice. 100 participants were initially recruited. Six participants were excluded from the final sample due to technical issues, leaving a final sample of 94 (69 women). For full details see Lehmann and colleagues^58^.

Informed consent was obtained from all participants prior to the experiments and they were compensated for their time (see Table 2).

**Table 2:** Differences between the three datasets in Study 1.

	Dataset 1	Dataset 2	Dataset 3
Sample size	85 (43 women)	121 (58 women)	89 (59 women)
Mean age (range)	YA: 24.0 (18–30) OA: 69.5 (65–77)	25.5 (18–57)	24.9 (18–65)
Original investigation	Age differences	Personality	Meditation/prosocial behaviour
Study design	Mixed	Within-subject	Within-subject
Previous publication	Stietz et al.^63^	Hildebrandt et al.^64^	Lehmann et al.^58^
Experiment location	MRI Scanner	MRI Scanner	Laboratory
Rating bar duration*	7 s	4 s	4 s
Question response duration^+^	25 s	15 s	15 s
Additional measures during original investigation	Socio-demographic questionnaire^1^ Cognitive battery^2,4^	Socio-demographic questionnaire^1^ 14-day ecological momentary assessment^3^ Unspecified online survey including trait measures of personality^3^ Interpersonal Reactivity Index^3^	Meditation training^3^
Compensation	€8.50	€120	€30 or course credit

*YA* younger adults, *OA* older adults.

*Duration the rating bar appeared on the screen during the EmpaToM task (empathy and compassion measures).

^+^Maximum duration for the ToM and factual reasoning questions during the EmpaToM task.

^1^Measure taken prior to the EmpaToM task.

^2^Measures counterbalanced against the EmpaToM task.

^3^Measures taken after the EmpaToM task.

^4^Including the Trail Making Test A and B, Identical Pictures Test, Digit Span Backward Test, and Spot a Word Test.

### EmpaToM

All three experiments used the validated EmpaToM task to measure empathy, compassion and ToM^3,66^. Across several previous behavioral and fMRI studies, validation of the EmpaToM task’s empathy, compassion, and ToM measures involved direct correlations and activation overlap with established empathy (Socio-affective Video Taks;^67^) and ToM tasks (False Belief Task;^68^, Imposing Memory Task;^69^), as well as exhibiting overlap with previous meta-analytical findings^67–70^.

**Figure 6:**
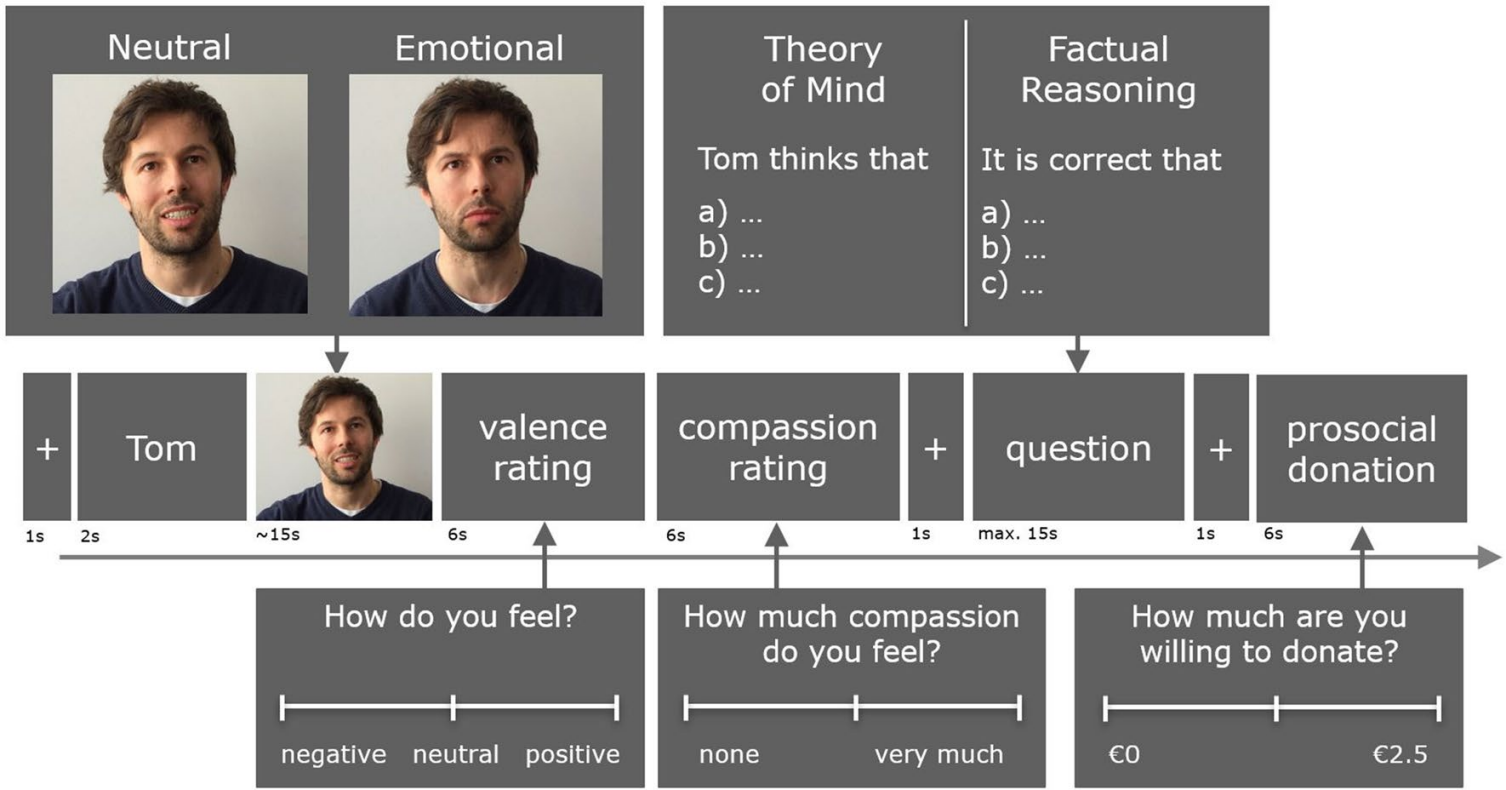
Example of an EmpaToM Task trial. The EmpaToM task utilizes a within-subject design. In each trial (48 in total), participants were shown either an emotionally negative or neutral video of a man or a woman describing an autobiographical experience. The content of the experience further required either factual-reasoning or ToM inference. Following the video, participants indicated on a continuous valence scale how they felt, that is, how much the participant shared the negative feelings expressed by the video’s protagonist (empathy measure). This was followed by an additional continuous scale on which participants indicated how much compassion they felt (compassion measure). Participants were then presented with a multiple-choice question (three choices, only one correct answer) that required either ToM inference or factual reasoning. Last, participants indicated on a continuous scale how much money they were willing to donate to a charitable cause associated with the content of the video (prosocial donation measure, Study 2 only). Timings indicated are for EmpaToM version used for Study 2. Differences in timing between the dataset versions for Study 1 can be found in Table 2. Informed consent was obtained to publish the individual’s images depicted in this figure.

The EmpaToM task utilizes a within-subject design and involves participants viewing a ~ 15 second video clip during each trial (48 in total), in which a narrator recounts an allegedly autobiographical experience with either neutral (e.g., moving into a new apartment) or negatively emotional content (e.g., relative with a disease). During each trial participants watched a video, after which they indicated the valence of their affective state on a continuous scale, with negative ratings after an emotionally negative narration indicating empathic sharing of the narrator’s emotion (empathy measure). Another continuous scale on which participants indicated how much compassion they felt for the person in the video (compassion measure) directly followed this. Participants were then presented with a multiple-choice question (three choices, one correct answer) that required either ToM inference (“The person thinks that…”) or factual-reasoning as a control condition (“It is correct that…”). Additionally in D3, participants reported their hypothetical willingness to help the narrator. However, due to the small sample size and, thus, insufficient statistical power, gender differences in this prosocial rating were not analysed for the current study. Before beginning the experiment, participants completed a practice round with instructions to familiarize themselves with the paradigm. The display timings of the rating bars and questions differed slightly between each dataset and are summarized in Table 2. For an illustrative example of the EmpaToM task, see Fig. 6 (note: informed consent was obtained to publish the individual’s images depicted in the figure). For examples of the videos’ content, see the supplementary material (S1: Example stories and questions for each experimental condition). Participants viewed 48 videos in total, across all datasets. The videos contained six different female and six different male narrators. The ordering of the videos were pseudo-randomized to control for frequency of male and female narrators, frequency of video’s valence or question type, and frequently of the narrator. The version of the EmpaToM task used across the three datasets was programed in Presentation (https://www.neurobs.com) and took approximately 45 min to complete.

### Procedure

The current study combines three summary datasets of the EmpaToM paradigm. The datasets were selected based on a review of the available, published online datasets for the EmpaToM task involving healthy adults. Three datasets fit this criteria and were available for download. The datasets were obtained from the online repository Open Science Framework. In Table 2, we summarize the datasets, including the additional measures that were not analysed in the current study. In each of the original investigations, participants completed the EmpaToM task alone, under laboratory conditions at the Technische Universität Dresden.

In brief, the procedures for the three original studies were as follows:

D1 (investigating age differences in social abilities): The experimental procedure involved two counterbalanced sessions in which participants either (i) completed the EmpaToM task during functional magnetic resonance imaging or (ii) undertook a battery of cognitive functioning tasks. At the beginning of the first session participants also completed a socio-demographic questionnaire^63^. Participants were compensated €8.50 for their time. The statistical analysis compared empathy, compassion, and ToM measures between younger and older adults in a mixed design.

D2 (investigating personality traits and social abilities): Participants were selected based on an online screening in which suitability for MRI scanning and demographics were assessed along with other questionnaires not reported in the original paper. The experimental procedure involved participants completing the EmpaToM task, combined with a subsequent 14-day ecological momentary assessment protocol on social interactions^64^. Participants were compensated €120 for their time. Statistical analysis compared both behavioural and neuronal measures collected during the EmpaToM task with everyday social affect and social cognition measures collected during the ecological momentary assessment in a with-in subject design.

D3 (investigating meditation practice, prosociality, and social abilities): The experimental procedure involved participants completing the EmpaToM task, with this data acquisition occurring at the start of a brief meditation training study^58^. Participants were compensated €30 or course credit for their time. Lehmann and colleges used this dataset to investigate statistical relationships between empathy, compassion, and theory of mind with the prosocial behaviour measure in a within subject design.

### Data

In the current study, we focus on the empathy, compassion and ToM measures of the EmpaToM task, comparing emotional with neutral videos and ToM with factual reasoning questions. Validation of these contrasts is described in detail by Kanske and colleagues^3^.The data used in the current study was summary data from the EmpaToM task. For each participant, an average was generated for each of the main measures. For empathy and compassion, this involved the average across the rating bar responses from the 24 emotional and 24 neutral videos. For the ToM measures, accuracy reflects the percentage of the 24 ToM and 24 factual reasoning questions correctly answered. RT is the corresponding average time taken to respond to these correctly answered ToM and factual reasoning questions.

## Methods: study 2

Study 2 was conducted online. The Ethics Committee of Technische Universität Dresden (EK-98022021) approved this study. All methods were performed in accordance with the relevant guidelines and regulations. The power analysis for Study 2 was same as in Study 1. Mann–Whitney U and ANOVA analyses were performed using SPSS (IBM Corp., 2021, Version 28.0.1.0). Correlation analyses and graphic production were done using Graphpad Prism (https://www.graphpad.com, Version 9.3.1). Generalized linear model analysis was performed using Stata (StataCorp, 2019, Version 15.1). The data and analysis scripts for Study 2 are available at https://osf.io/xgma6/?view_only=d0b7d3e1f10a415fa6c033678de5c9ff. The analysis for the current study was not preregistered.

### Participants

The EmpaToM data used in the current study is derived from a separate investigation examining creativity and social behaviour. The pre-registration for this original study is available at https://aspredicted.org/blind.php?x=2vk3a6. Please note that the participant exclusion criteria for the current analyses does not deviate from the pre-registration for the original dataset investigating creativity and social behaviour. The original creativity study utilized a within-subject design.

Participants were recruited via Prolific (www.prolific.co) and tested in the context of an online study examining creativity and social abilities. To account for potential dropouts and other issues, 250 participants were invited to take part. Of these, 23 participants were excluded due to technical issues or not completing the experiment (7), using an unsupported browser or operating system (6), answering less than 33% of the questions correctly or answering on average in less than 1.5 s (6), limited or no rating bar movement (2), and experimenter error (2). In addition, multivariate outliers were screened for among social affect, social cognition, and demographic variables, generating Mahalanobis distance scores from a multiple linear regression. For the current dataset, there were 13 degrees of freedom, which equated to a critical Chi-square value of 34.529 (at α = 0.001). The test revealed one multivariate outlier that was additionally removed from the analyses. Thus, 226 individuals (101 women) were included in the final dataset. A Mann–Whitney U test indicated that men (age mean = 30.02, SD = 7.75) and women (age mean = 32.59, SD = 12.53) did not differ significantly in age (U = 6235, p = 0.87). Participants reported their gender (man, woman, or diverse), age, and highest obtained level of education. None of the participants indicated that they identify as diverse. Highest obtained level of education was indicated based on 7 possible answers, conforming to the German education system, which was then concatenated into three categories: high school (German: Hauptschule, Realschule, Fachhochschule- oder allg. Hochschulreife), vocational training (German: Berufsausbildung), and university (German: Bachelor, Master, Promotion). In total, 14 participants reported high school education, 100 participants reported vocational training, and 112 participants reported a university education as their highest obtained level of education. Demographic data were collected via FormR^71^. Informed consent was obtained from participants prior to the experiment. Participants were debriefed as to the intention of the experiment at the end of data collection and received €12 reimbursement for their time.

### EmpaToM

To allow for online testing, the original, offline version of the EmpaToM was translated into JavaScript using the PsychoJS library (https://github.com/psychopy/psychojs) and presented online via Pavlovia^72^. The online version of the EmpaToM takes approximately 40 min to complete. The empathy, compassion and ToM/factual reasoning measures of the EmpaToM task were the same in Study 2 as in Study 1 with the timing of each measure indicated in Fig. 1. In addition, participants in Study 2 answered a prosocial behaviour question integrated into the EmpaToM by combining it with the option to engage in a charitable donation (see^73,27^). For the prosocial donation question, participants were endowed with €2.50 per trial. They could donate none, part, or this entire amount to a charity associated with each video. Specifically, during the instructions, participants were informed that a charity, thematically appropriate to the content of each video’s narrative (e.g., *Red Cross* for a narrative discussing homelessness, *German Cancer Aid* for a narrative discussing a mother with cancer, etc.), had been selected and that they could donate up to €2.50 per trial to that given charity. Participants were then informed that a single trial would be randomly selected at the end of the experiment and the amount they chose to give during this single, random trial would be donated to the charity associated with the selected video. They were also told that the money they chose not to donate would be paid to them in addition to their compensation. Thus, the prosocial donation measure used in Study 2 was associated with a real-world cost for the participants.

### Procedure

For Study 2, participants were invited online via Prolific to take part in a study exploring social behaviour. After initial invitation via Prolific, participants received a link to the FormR experiment page. The link was only valid with the access code and became invalid once a participant completed the study, thus limiting the possibility that participants would undertake the study twice. After accessing the link, participants were required to complete a series of questionnaires via FormR. These included a demographic questionnaire the Alternate Use Task and Instances Task^74^, Inventory of Creative Activities and Achievements^74,75^, Author Recognition Test^76^, IQ measures (WAIS IV Matrix Reasoning Task and Mehrfachwahl-Wortschatz-Intelligenztest) and standard personality measures (Big Five Inventory and Interpersonal Reactivity Index). Only the demographic questionnaire was used in the current study. On finishing the final questionnaire, participants were then transferred to the Pavlovia experiment webpage where they undertook the online version of the EmpaToM paradigm. Participants were compensated with €12.50 for their time.

### Data

The empathy, compassion and ToM measures used in in Study 2 are same as described in the Data section in Study 1. For the prosocial donations measure, the average donation for each participant across the 24 emotional and 24 neutral videos was used.

### Supplementary Information

Supplementary Tables.

## Data Availability

The data that support the findings of this study are openly available at the Open Science Framework: *Study 1* Dataset 1: https://doi.org/10.17605/OSF.IO/7EDBN. Dataset 2 https://osf.io/f7zcr/. Dataset 3 https://osf.io/tu2gj/?view_only=f285308c035d451ca2fce5f3788f97e3. *Study 2* (including full dataset and analysis scripts for both studies). https://osf.io/xgma6/?view_only=d0b7d3e1f10a415fa6c033678de5c9ff.
