# Supplementary material for: Gender differences in empathy, compassion, and prosocial donations, but not theory of mind in a naturalistic social task
Source: Sci Rep. 2023 Nov 25;13:20748. doi: 10.1038/s41598-023-47747-9 (PMC10676355; doi:10.1038/s41598-023-47747-9)
Supplement: Supplementary file 1 — Supplementary Tables. [file 41598_2023_47747_MOESM1_ESM.pdf]

Gender differences in empathy, compassion, and prosocial donations, but not theory of mind in a naturalistic social task: Supplemental Material and Data

Supplement S1: Example stories and questions for each experimental condition

Example 1: Anna

Neutral nonToM

„Hm… well, … that evening, I cooked. I prepared one of those 3-course meals. And my boyfriend invited his sister. She brought a nice red wine from her vacation in France. And then there was my former flatmate. Well, I think we were sitting in the kitchen until one, one thirty."

It is true

- that Anna's boyfriend was at the party. **(correct)**
- that Anna has been to France before and has brought some red wine.
- that Anna has been living with friends for quite a while.

Neutral ToM

„My best friend Laura recently went to the movies with my brother. And she loves these cartoon movies, and my brother also thought the movie was great. He wants to watch another one of those with her right next week. That doesn't really sound like him… he used to be more into the action stuff."

Anna thinks

- that her brother fell in love with her best friend and this is why he watches cartoon movies with her. **(correct)**
- that her brother's being in love entirely changed his taste in movies.
- that her brother plans to also watch action movies with her best friend.

Emotional nonToM

„We've been together for five years now, and it wasn't like we didn't like each other any more. But… at some point we just couldn't stop fighting. And once, he got so mad at me, he… hit me in the face. I just couldn't really go on after that."

It is true

- that Anna met her ex-boyfriend at least five years ago. **(correct)**
- that Anna and her boyfriend don't see each other often since they started dating.
- that Anna's ex-boyfriend was often violent.

Emotional ToM

„My sister was diagnosed with bowel cancer a year ago and the odds aren't great. But you have to cling to something, don't you. Her doctor recently suggested a new treatment to her, but she refuses to try. It just makes me wanna cry."

Anna thinks

- that her sister gave up hope and doesn't want treatment anymore. **(correct)**
- that her sister wants to look for an appropriate treatment herself.
- that her sister would probably be saved by the new treatment.

Example 2: Hannes

Neutral nonToM

„I am doing quite a lot of reading at the moment, mainly German classics, Thomas Mann, for example. I like it and I also need it for a class. And when I am through, I want to dive into Russian literature."

It is true

- that Hannes has read more German than Russian literature in the past. **(correct)**
- that Hannes needs to read a lot for his thesis.
- that Hannes studies philosophy, which is why he needs to read all the classics.

Neutral ToM

„It was a gift, Katharina gave me these ridiculously expensive musical tickets. I sold them, but told her that I really enjoyed the show. When I did, somehow her smile froze … "

Hannes thinks

- that Katharina wanted to go to the musical together with him. **(correct)**
- that Katharina does not believe he liked the musical so much..
- that he would have really liked the musical.

Emotional nonToM

„I got beaten up in the subway pretty bad, by three guys. They circled me and kicked me… And nobody helped."

It is true

- that Hannes could not run from the offenders. **(correct)**
- that Hannes had bumped into the men in the subway and then they beat him up.
- that Hannes screamed for help, when the men beat him up.

Emotional ToM

„My father is an alcoholic. Often times he doesn't come home for days and then the police brings him. My mom is worried like crazy. And I am also worried, but I cannot come home each time ..."

Hannes thinks

- that his mother expects him to take better care of her. **(correct)**
- that his father knows how much his mother is worried.
- that his father will always find his way back home eventually.

Study 1: Empathy

Test of within-subject effects

Supplementary Table 1

Source		**Type III sum of squares**	df	Mean Square	F	Sig.	**Partial Eta Squared**
Empathy	Sphericity assumed	114,076	1	114,076	358,874	<,001	,552
	Greenhouse-Geisser	114,076	1,000	114,076	358,874	<,001	,552
	Huynh-Feldt	114,076	1,000	114,076	358,874	<,001	,552
	Lower Bound	114,076	1,000	114,076	358,874	<,001	,552
Empathy * Age	Sphericity assumed	,241	1	,241	,759	,384	,003
	Greenhouse-Geisser	,241	1,000	,241	,759	,384	,003
	Huynh-Feldt	,241	1,000	,241	,759	,384	,003
	Lower Bound	,241	1,000	,241	,759	,384	,003
Empathy * Experimental Location	Sphericity assumed	,250	1	,250	,787	,376	,003
	Greenhouse-Geisser	,250	1,000	,250	,787	,376	,003
	Huynh-Feldt	,250	1,000	,250	,787	,376	,003
	Lower Bound	,250	1,000	,250	,787	,376	,003
Empathy * Gender	Sphericity assumed	2,977	1	2,977	9,364	,002	,031
	Greenhouse-Geisser	2,977	1,000	2,977	9,364	,002	,031
	Huynh-Feldt	2,977	1,000	2,977	9,364	,002	,031
	Lower Bound	2,977	1,000	2,977	9,364	,002	,031
Error(Empathy)	Sphericity assumed	92,500	291	,318			
	Greenhouse-Geisser	92,500	291,000	,318			
	Huynh-Feldt	92,500	291,000	,318			
	Lower Bound	92,500	291,000	,318			

Test of between-subject effects

Source	Type III sum of squares	df	Mean Square	F	Sig.	Partial Eta Squared
Constant Term	39,008	1	39,008	138,050	<,001	,322
Age	2,225	1	2,225	7,876	,005	,026
Experimental Location	2,591	1	2,591	9,169	,003	,031
Gender	3,347	1	3,347	11,845	<,001	,039
Error	82,226	291	,283			

Study 1: Compassion

Test of within-subject effects

Supplementary Table 2

Source		Type III sum of squares	df	Mean Square	F	Sig.	Partial Eta Squared
Compassion	Sphericity assumed	165,718	1	165,718	328,242	<,001	,530
	Greenhouse-Geisser	165,718	1,000	165,718	328,242	<,001	,530
	Huynh-Feldt	165,718	1,000	165,718	328,242	<,001	,530
	Lower Bound	165,718	1,000	165,718	328,242	<,001	,530
Compassion* Age	Sphericity assumed	5,514	1	5,514	10,922	,001	,036
	Greenhouse-Geisser	5,514	1,000	5,514	10,922	,001	,036
	Huynh-Feldt	5,514	1,000	5,514	10,922	,001	,036
	Lower Bound	5,514	1,000	5,514	10,922	,001	,036
Compassion * Experimental Location	Sphericity assumed	,172	1	,172	,340	,560	,001
	Greenhouse-Geisser	,172	1,000	,172	,340	,560	,001
	Huynh-Feldt	,172	1,000	,172	,340	,560	,001
	Lower Bound	,172	1,000	,172	,340	,560	,001
Compassion * Gender	Sphericity assumed	3,834	1	3,834	7,595	,006	,025
	Greenhouse-Geisser	3,834	1,000	3,834	7,595	,006	,025
	Huynh-Feldt	3,834	1,000	3,834	7,595	,006	,025
	Lower Bound	3,834	1,000	3,834	7,595	,006	,025
Error(Compassion)	Sphericity assumed	146,915	291	,505			
	Greenhouse-Geisser	146,915	291,000	,505			
	Huynh-Feldt	146,915	291,000	,505			
	Lower Bound	146,915	291,000	,505			

Test of between-subject effects

Source	Type III sum of squares	df	Mean Square	F	Sig.	Partial Eta Squared
Constant Term	1028,307	1	1028,307	966,380	<,001	,769
Age	47,913	1	47,913	45,028	<,001	,134
Experimental Location	8,721	1	8,721	8,196	,005	,027
Gender	11,047	1	11,047	10,382	,001	,034
Error	309,648	291	1,064			

Study 1: Question Accuracy

Test of within-subject effects

Supplementary Table 3

Source		**Type III sum of squares**	df	Mean Square	F	Sig.	**Partial Eta Squared**
Accuracy	Sphericity assumed	151,418	1	151,418	1,636	,202	,006
	Greenhouse-Geisser	151,418	1,000	151,418	1,636	,202	,006
	Huynh-Feldt	151,418	1,000	151,418	1,636	,202	,006
	Lower Bound	151,418	1,000	151,418	1,636	,202	,006
Accuracy * Age	Sphericity assumed	3752,196	1	3752,196	40,542	<,001	,122
	Greenhouse-Geisser	3752,196	1,000	3752,196	40,542	<,001	,122
	Huynh-Feldt	3752,196	1,000	3752,196	40,542	<,001	,122
	Lower Bound	3752,196	1,000	3752,196	40,542	<,001	,122
Accuracy * Experimental Location	Sphericity assumed	1,077	1	1,077	,012	,914	,000
	Greenhouse-Geisser	1,077	1,000	1,077	,012	,914	,000
	Huynh-Feldt	1,077	1,000	1,077	,012	,914	,000
	Lower Bound	1,077	1,000	1,077	,012	,914	,000
Accuracy * Gender	Sphericity assumed	118,654	1	118,654	1,282	,258	,004
	Greenhouse-Geisser	118,654	1,000	118,654	1,282	,258	,004
	Huynh-Feldt	118,654	1,000	118,654	1,282	,258	,004
	Lower Bound	118,654	1,000	118,654	1,282	,258	,004
Error(Accuracy)	Sphericity assumed	26932,461	291	92,551			
	Greenhouse-Geisser	26932,461	291,000	92,551			
	Huynh-Feldt	26932,461	291,000	92,551			
	Lower Bound	26932,461	291,000	92,551			

Test of between-subject effects

Source	Type III sum of squares	df	Mean Square	F	Sig.	Partial Eta Squared
Constant Term	725246,788	1	725246,788	3025,227	<,001	,912
Age	36695,011	1	36695,011	153,066	<,001	,345
Experimental Location	2597,667	1	2597,667	10,836	,001	,036
Gender	1150,926	1	1150,926	4,801	,029	,016
Error	69762,297	291	239,733			

Study 1: Question Reaction Times

Test of within-subject effects

Supplementary Table 4

Source		**Type III sum of squares**	df	Mean Square	F	Sig.	**Partial Eta Squared**
RT	Sphericity assumed	3,409	1	3,409	10,229	,002	,034
	Greenhouse-Geisser	3,409	1,000	3,409	10,229	,002	,034
	Huynh-Feldt	3,409	1,000	3,409	10,229	,002	,034
	Lower Bound	3,409	1,000	3,409	10,229	,002	,034
RT * Age	Sphericity assumed	2,771	1	2,771	8,315	,004	,028
	Greenhouse-Geisser	2,771	1,000	2,771	8,315	,004	,028
	Huynh-Feldt	2,771	1,000	2,771	8,315	,004	,028
	Lower Bound	2,771	1,000	2,771	8,315	,004	,028
RT * Experimental Location	Sphericity assumed	,167	1	,167	,501	,480	,002
	Greenhouse-Geisser	,167	1,000	,167	,501	,480	,002
	Huynh-Feldt	,167	1,000	,167	,501	,480	,002
	Lower Bound	,167	1,000	,167	,501	,480	,002
RT * Gender	Sphericity assumed	,188	1	,188	,565	,453	,002
	Greenhouse-Geisser	,188	1,000	,188	,565	,453	,002
	Huynh-Feldt	,188	1,000	,188	,565	,453	,002
	Lower Bound	,188	1,000	,188	,565	,453	,002
Error(RT)	Sphericity assumed	96,991	291	,333			
	Greenhouse-Geisser	96,991	291,000	,333			
	Huynh-Feldt	96,991	291,000	,333			
	Lower Bound	96,991	291,000	,333			

Test of between-subject effects

Source	Type III sum of squares	df	Mean Square	F	Sig.	Partial Eta Squared
Constant Term	5209,632	1	5209,632	764,023	<,001	,724
Age	190,145	1	190,145	27,886	<,001	,087
Experimental Location	56,227	1	56,227	8,246	,004	,028
Gender	20,698	1	20,698	3,036	,083	,010
Error	1984,237	291	6,819			

Study 2: Empathy

Test of within-subject effects

Supplementary Table 5

Source		**Type III sum of squares**	df	Mean Square	F	Sig.	**Partial Eta Squared**
Empathy	Sphericity assumed	39,142	1	39,142	87,505	<,001	,282
	Greenhouse-Geisser	39,142	1,000	39,142	87,505	<,001	,282
	Huynh-Feldt	39,142	1,000	39,142	87,505	<,001	,282
	Lower Bound	39,142	1,000	39,142	87,505	<,001	,282
Empathy * Age	Sphericity assumed	,000	1	,000	,000	,987	,000
	Greenhouse-Geisser	,000	1,000	,000	,000	,987	,000
	Huynh-Feldt	,000	1,000	,000	,000	,987	,000
	Lower Bound	,000	1,000	,000	,000	,987	,000
Empathy * Gender	Sphericity assumed	1,923	1	1,923	4,298	,039	,019
	Greenhouse-Geisser	1,923	1,000	1,923	4,298	,039	,019
	Huynh-Feldt	1,923	1,000	1,923	4,298	,039	,019
	Lower Bound	1,923	1,000	1,923	4,298	,039	,019
Error(Empathy)	Sphericity assumed	99,750	223	,447			
	Greenhouse-Geisser	99,750	223,000	,447			
	Huynh-Feldt	99,750	223,000	,447			
	Lower Bound	99,750	223,000	,447			

Test of between-subject effects

Source	Type III sum of squares	df	Mean Square	F	Sig.	Partial Eta Squared
Constant Term	21,267	1	21,267	98,302	<,001	,306
Age	,003	1	,003	,013	,908	,000
Gender	3,681	1	3,681	17,013	<,001	,071
Error	48,244	223	,216			

Study 2: Compassion

Test of within-subject effects

Supplementary Table 6

Source		**Type III sum of squares**	df	Mean Square	F	Sig.	**Partial Eta Squared**
Compassion	Sphericity assumed	62,676	1	62,676	86,265	<,001	,279
	Greenhouse-Geisser	62,676	1,000	62,676	86,265	<,001	,279
	Huynh-Feldt	62,676	1,000	62,676	86,265	<,001	,279
	Lower Bound	62,676	1,000	62,676	86,265	<,001	,279
Compassion* Age	Sphericity assumed	,172	1	,172	,236	,627	,001
	Greenhouse-Geisser	,172	1,000	,172	,236	,627	,001
	Huynh-Feldt	,172	1,000	,172	,236	,627	,001
	Lower Bound	,172	1,000	,172	,236	,627	,001
Compassion * Gender	Sphericity assumed	3,803	1	3,803	5,235	,023	,023
	Greenhouse-Geisser	3,803	1,000	3,803	5,235	,023	,023
	Huynh-Feldt	3,803	1,000	3,803	5,235	,023	,023
	Lower Bound	3,803	1,000	3,803	5,235	,023	,023
Error(Compassion)	Sphericity assumed	162,023	223	,727			
	Greenhouse-Geisser	162,023	223,000	,727			
	Huynh-Feldt	162,023	223,000	,727			
	Lower Bound	162,023	223,000	,727			

Test of between-subject effects

Source	Type III sum of squares	df	Mean Square	F	Sig.	Partial Eta Squared
Constant Term	329,232	1	329,232	167,204	<,001	,429
Age	1,898	1	1,898	,964	,327	,004
Gender	18,861	1	18,861	9,579	,002	,041
Error	439,097	223	1,969			

Study 2: Question Accuracy

Test of within-subject effects

Supplementary Table 7

Source		**Type III sum of squares**	df	Mean Square	F	Sig.	**Partial Eta Squared**
Accuracy	Sphericity assumed	1293,949	1	1293,949	12,065	<,001	,051
	Greenhouse-Geisser	1293,949	1,000	1293,949	12,065	<,001	,051
	Huynh-Feldt	1293,949	1,000	1293,949	12,065	<,001	,051
	Lower Bound	1293,949	1,000	1293,949	12,065	<,001	,051
Accuracy * Age	Sphericity assumed	10,738	1	10,738	,100	,752	,000
	Greenhouse-Geisser	10,738	1,000	10,738	,100	,752	,000
	Huynh-Feldt	10,738	1,000	10,738	,100	,752	,000
	Lower Bound	10,738	1,000	10,738	,100	,752	,000
Accuracy * Gender	Sphericity assumed	19,812	1	19,812	,185	,668	,001
	Greenhouse-Geisser	19,812	1,000	19,812	,185	,668	,001
	Huynh-Feldt	19,812	1,000	19,812	,185	,668	,001
	Lower Bound	19,812	1,000	19,812	,185	,668	,001
Error(Accuracy)	Sphericity assumed	23916,758	223	107,250			
	Greenhouse-Geisser	23916,758	223,000	107,250			
	Huynh-Feldt	23916,758	223,000	107,250			
	Lower Bound	23916,758	223,000	107,250			

Test of between-subject effects

Source	Type III sum of squares	df	Mean Square	F	Sig.	Partial Eta Squared
Constant Term	186690,105	1	186690,105	730,012	<,001	,766
Age	132,514	1	132,514	,518	,472	,002
Gender	348,779	1	348,779	1,364	,244	,006
Error	57029,060	223	255,736			

Study 2: Question Reaction Times

Test of within-subject effects

Supplementary Table 8

Source		**Type III sum of squares**	df	Mean Square	F	Sig.	**Partial Eta Squared**
RT	Sphericity assumed	,379	1	,379	1,457	,229	,006
	Greenhouse-Geisser	,379	1,000	,379	1,457	,229	,006
	Huynh-Feldt	,379	1,000	,379	1,457	,229	,006
	Lower Bound	,379	1,000	,379	1,457	,229	,006
RT * Age	Sphericity assumed	,002	1	,002	,006	,937	,000
	Greenhouse-Geisser	,002	1,000	,002	,006	,937	,000
	Huynh-Feldt	,002	1,000	,002	,006	,937	,000
	Lower Bound	,002	1,000	,002	,006	,937	,000
RT * Gender	Sphericity assumed	,116	1	,116	,445	,505	,002
	Greenhouse-Geisser	,116	1,000	,116	,445	,505	,002
	Huynh-Feldt	,116	1,000	,116	,445	,505	,002
	Lower Bound	,116	1,000	,116	,445	,505	,002
Error(RT)	Sphericity assumed	58,009	223	,260			
	Greenhouse-Geisser	58,009	223,000	,260			
	Huynh-Feldt	58,009	223,000	,260			
	Lower Bound	58,009	223,000	,260			

Test of between-subject effects

Source	Type III sum of squares	df	Mean Square	F	Sig.	Partial Eta Squared
Constant Term	1957,861	1	1957,861	649,861	<,001	,745
Age	6,216	1	6,216	2,063	,152	,009
Gender	16,583	1	16,583	5,504	,020	,024
Error	671,841	223	3,013			

Supplementary Table 9. Summary of Generalized Linear Model for Variables Predicting Prosocial Donations (N = 226)

	**exp(b)**	**Robust St. Error**	**z-value**	**CI-95%**	**p-value**
Constant	.402	.425	-.86	[.05 - 3.19]	.390
ToM Accuracy	.977	.013	-1.63	[.95 - 1.00]	.104
Factual Accuracy	1.02	.011	1.80	[.99 - 1.09]	.072
Age	1.00	.010	0.15	[.98 - 1.02]	..882
Level of Education					
Reference Level - High School					
Vocational Training	1.05	.475	0.11	[.43 - 2.54]	.912
University	1.08	.477	0.17	[.45 - 2.56]	.862
Gender					
Reference Level - Men					
Women	.188	..260	-1.21	[.01 - 2.83]	.228
Interactions					
ToM Accuracy x Gender	1.05	.021	2.48	[1.01 - 1.09]	**.013***
Factual Accuracy x Gender	.978	.015	-1.43	[.94 - 1.00]	.154

Note: Full Stata output available at https://osf.io/xgma6

Supplementary Table 10. Interaction effect gender x ToM accuracy predicting donation amount in men and women

	**ey/dx**	**Std. Err.**	**z-value**	**CI-95%**	**p-value**
Men	-.022	.015	-1.50	[-.052 - .006]	0.13
Women	.027	.013	2.06	[.001- .054]	**0.04***

Note: Full Stata output available at https://osf.io/xgma6
